# Dysregulation of miRNAs in bladder cancer: altered expression with aberrant biogenesis procedure

**DOI:** 10.18632/oncotarget.15173

**Published:** 2017-02-07

**Authors:** Fan Dong, Tianyuan Xu, Yifan Shen, Shan Zhong, Shanwen Chen, Qiang Ding, Zhoujun Shen

**Affiliations:** ^1^ Department of Urology, Fudan Institute of Urology, Huashan Hospital, Fudan University, Shanghai, China; ^2^ Department of Urology, Ruijin Hospital, School of Medicine, Shanghai Jiaotong University, Shanghai, China

**Keywords:** microRNA, bladder cancer, expression profile, biogenesis procedure

## Abstract

Aberrant expression profiles of miRNAs are widely observed in the clinical tissue specimens and urine samples as well as the blood samples of bladder cancer patients. These profiles are closely related to the pathological features of bladder cancer, such as the tumour stage/grade, metastasis, recurrence and chemo-sensitivity. MiRNA biogenesis forms the basis of miRNA expression and function, and its dysregulation has been shown to be essential for variations in miRNA expression profiles as well as tumourigenesis and cancer progression. In this review, we summarize the up-to-date and widely reported miRNAs in bladder cancer that display significantly altered expression. We then compare the miRNA expression profiles among three different sample types (tissue, urine and blood) from patients with bladder cancer. Moreover, for the first time, we outline the dysregulated miRNA biogenesis network in bladder cancer from different levels and analyse its possible relationship with aberrant miRNA expression and the pathological characteristics of the disease.

## INTRODUCTION

Urinary bladder cancer (BCa), which ranks number seven on the list of the most common malignancies in male patients, severely impairs public health. The morbidity and mortality of bladder cancer are both the second-highest among all urinary tumours, just after prostate cancer [1]. Although the majority of patients (75%-85%) suffer from non-muscle invasive bladder cancer (NMIBC) (pTa-pT1), the recurrence rate following the transurethral resection of bladder tumours (TURBT) can be up to 75% within 5 years [2], and the disease may invade the muscle layer very quickly. Bladder tumours are not easy to cure due to their high recurrence and metastasis rates, with a five-year survival rate of approximately 57% [3]. Thus, research on BCa, especially on the mechanisms of carcinogenesis and tumour progression, still need more time to evolve. New discoveries are required to update the diagnostic methods as well as the therapeutic strategies.

MicroRNAs (miRNAs) are a group of highly conserved small non-coding RNAs [4], and they can post-transcriptionally regulate gene expression by directly targeting mRNAs. They are widely involved in the proliferation, differentiation and apoptosis of cells [5]. MiRNAs may work as either oncogenes or tumour suppressors, and they are deeply implicated in the oncogenesis and progression of various carcinomas, including bladder cancer [6-8]. A large number of studies have shown that the miRNA expression profile is significantly changed not only in tumour specimens but also in the urine and blood of bladder cancer patients [9-11]. Dysregulations in miRNA expression are closely related to the development of bladder cancer, such as chemotherapy resistance and a worse prognosis [12, 13].

MiRNA biogenesis has been shown to play an important role in bladder cancer. MiRNA biogenesis is a complicated network that starts from the transcription of coding genes and ends up with mature miRNAs targeting mRNAs [14]. Numerous proteins, enzymes and factors are involved in the regulation of miRNA biogenesis, including some key components such as DROSHA/DGCR8 [15] and DICER [16]. Recent research has confirmed that the abnormalities of the miRNA biogenesis pathway may alter the miRNA expression profile and have an impact on the progression of various urinary cancers [17, 18]. In bladder cancer, the theory above is further verified; that is, impaired miRNA biogenesis can directly induce the dysregulation and malfunction of different miRNAs, which may contribute to the clinical progression of bladder cancer [19, 20].

In this review, we summarize the current studies on changes in miRNA expression in bladder tumour tissues and urine samples as well as blood collected from patients, and we analyse the aberrantly regulated biogenesis network of BCa-related miRNAs from different angles. We deeply discuss their dysregulations in terms of coding genes, primary transcript processing, pre-miRNA maturation, the miRNA-mRNA binding process and the core components in miRNA biogenesis machinery. In this way, we reveal that an impaired biogenesis network in bladder cancer may lead to wide changes in miRNAs and eventually induce carcinogenesis and the development of BCa.

## BIOGENESIS PROCEDURE OF MIRNAS

**Figure 1:**
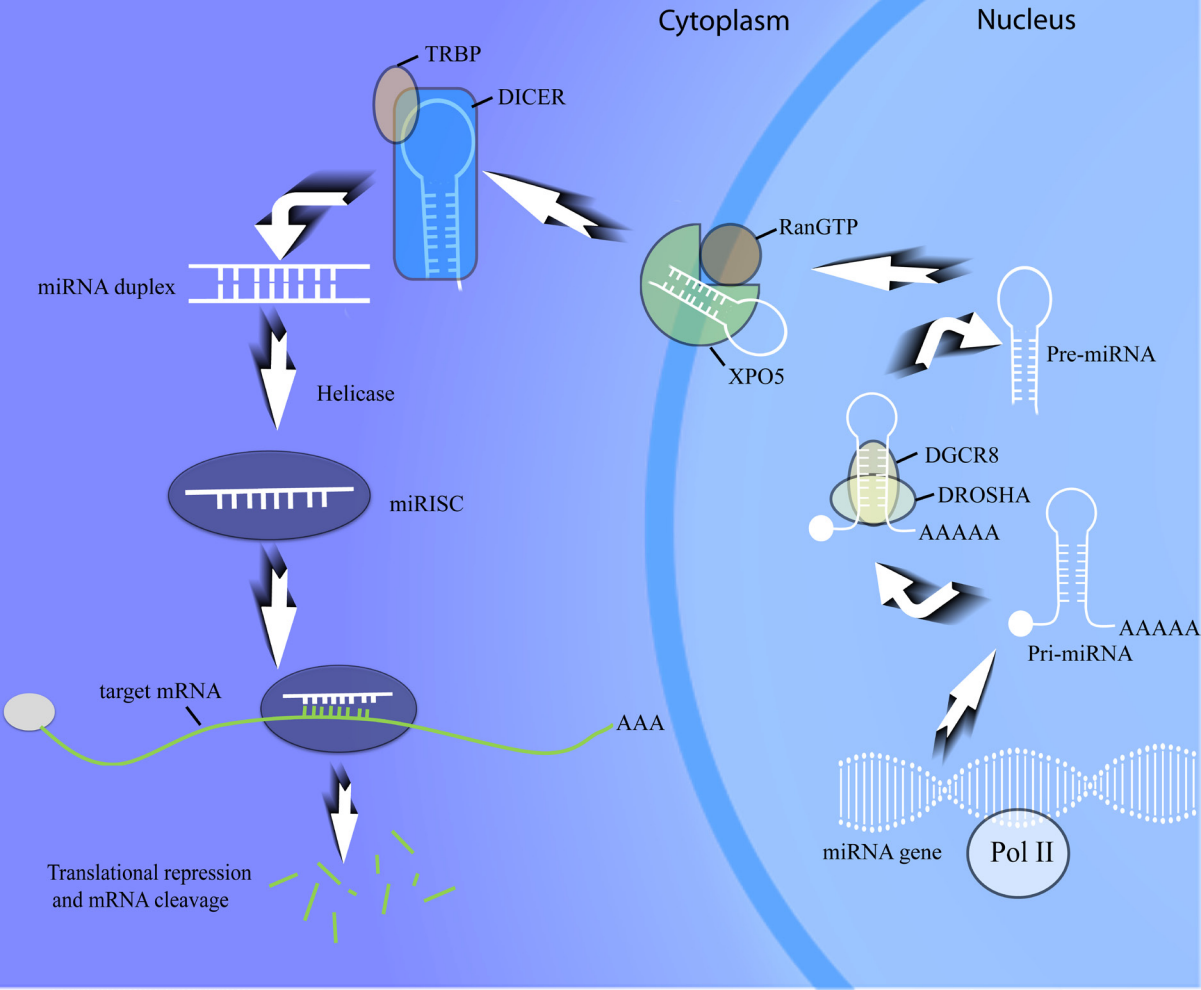
MiRNA biogenesis procedure The whole biological process basically includes coding gene transcription, pri-miRNA cleavage, pre-miRNA maturation, miRNA-mRNA binding and target mRNA degeneration. miRISC, miRNA-induced silencing complex; TRBP, transactivation-responsive RNA-binding protein.

The biogenesis pathway of miRNAs begins with the transcription of coding genes by RNA polymerase II (Figure 1). After the primary transcript (pri-miRNA) is generated, the pri-miRNA is then processed into a short precursor miRNA (pre-miRNA). This step is performed by Microprocessor, which contains an RNAse II enzyme (DROSHA) and DiGeorge syndrome critical region 8 (DGCR8) [15]. Pre-miRNA is next pumped out of the nucleus by XPO5 (Exportin 5) with the help of RanGTP. The RNAse III enzyme DICER, which is stored in the cytoplasm, will then cleave the pre-miRNA and generate a miRNA duplex. The guide strand of mature miRNA is installed in the miRNA-induced silencing complex (miRISC) and leads to binding with the miRNA binding site located in the 3'UTR of target mRNA. Afterwards, the translation of the target mRNA is suppressed and the corresponding gene is silenced. Considering that the mature miRNA would not work if there was any disorder in the miRNA-mRNA binding, we naturally count the step of “miRNA-mRNA binding” as the very last step of the entire miRNA biogenesis pathway, in accordance with previous studies [11, 21]. For further information about the miRNA biogenesis pathway, please refer to ref. 22 [22].

## SIGNIFICANT EXPRESSION CHANGES IN THE MIRNAS OF BLADDER CANCER

Hundreds of miRNAs are found to be aberrantly expressed in bladder cancer. In 2007, Gottardo *et al*. first reported on a set of miRNAs, including miR-223, miR-26b, miR-221, miR-103-1, miR-185, miR-23b, miR-203, miR-17-5p, miR-23a, and miR-205, that are dysregulated in bladder cancer. They analysed the involvement of these miRNAs in the development of the disease [23]. Since then, the disorders of the miRNA expression profile have been revealed gradually. A part of those changed miRNAs is characterized by downregulation while the other part remains over-expressed, compared with normal tissues. The role that a certain miRNA plays in bladder cancer may be reflected by its variation. For example, Ichimi screens fourteen BCas, five normal bladder urothelia samples and three BC cell lines to find that the expression levels of 19 out of 156 miRNAs decreased, and a subset of 4 miRNAs (miR-30-3p, miR-133a, miR-195 and miR-199a*), which usually act as tumour suppressors, are then validated as being downregulated in tumours [24]. By contrast, some other miRNAs such as miR-200c and miR-21 are obviously over-expressed in clinical BCa tissues and may have a function in promoting the development of bladder cancer [25, 26]. Numerous investigations using different types of biological samples (clinical tissue specimens, body fluids, bladder cancer cell lines, etc.) portray the general expression profile of miRNAs in the context of bladder cancer [9, 10, 24]. To have an insightful understanding of this field, a global review on the current literature about the dysregulations of miRNAs in bladder cancer is undertaken in this manuscript. Literature searches in PubMed for the MESH terms “bladder cancer” and “miRNA” retrieved 473 manuscripts between the years 2006 and 2017. The primary articles and their relevant references were both reviewed. We further summarize the findings on the miRNAs that are widely reported (at least in 2 manuscripts) to be aberrantly expressed in the following three types of biological samples: BCa tissue, urine and blood (Table 1). Studies that focus on either general miRNA expression changes or changes in a specific miRNA are all included in this review. MiRNAs that are reported to be dysregulated only by a single paper or in research that was only based on BCa cell lines are not counted.

**Table 1:** Dysregulated miRNAs among different biological samples reported by multiple studies in bladder cancer

miRNAs	Sample types	Up/Down	References	miRNAs	Sample types	Up/Down	References
let-7a	Tissue	↓	[9, 110]	let-7b	Tissue,Urine	↑,↑^U^	[27]/[39]U
let-7c	Tissue	↓	[9, 111-113]	miR-1	Tissue,Urine,Blood	↓,↓^U^,↓^B^	[9,27,28, 37,111,112, 114,115]/ [116]T&U/[117] B
miR-9	Tissue	↑	[37, 118, 119]	miR-10a	Tissue	↑	[27, 28, 114, 120]
miR-10b	Tissue,Urine	↑,↑^U^	[114, 120, 121]/[122]U	miR-15a a	Tissue,Urine	↑,↓^Ua^,↑^Ua^	[27]/[39, 40]U
miR-15b	Urine,Blood	↓^U^,↓^B^	[40]U/[123]B	miR-16	Tissue,Urine	↑,↑^U^	[27, 124]/[116]T&U
miR-17-5p	Tissue	↑	[9, 23, 42, 125]	miR-18a	Tissue, Blood	↑↑^B^	[9, 42]/[10]B
miR-18a*	Tissue,Urine	↑,↑^U^	[27]/[126]U	miR-19a	Tissue,Blood	↑,↑^B^	[9, 27]/[127]B/[128]T&B
miR-19b	Tissue	↑	[9, 27]	miR-20a	Tissue	↑	[9, 33, 125, 129]
miR-21	Tissue,Urine, Blood	↑,↑^U^,↑^B^	[26,27,30,120, 130, 131]/ [39,132]U/[10]U&B	miR-23b	Tissue	↓	[109, 131, 133]
miR-24-1	Tissue,Urine	↓,↓^U^	[40]U/[134]	miR-25	Tissue,Urine,Blood	↓,↑^U^,↓^B^	[27, 28, 42]/[126]U/[135] B
miR-26a	Tissue	↓	[34, 35, 110, 129]	miR-26b-5p	Tissue,Blood	↓,↑^B^	[10]B/[136]
miR-27a	Urine,Blood	↓^U^,↓^B^	[126]U/[123, 127]B	miR-27b	Tissue,Urine	↓,↓^U^	[40]U/[109]
miR-29a	Tissue,Urine	↓,↓^U^	[42, 129]/[10]U	miR-29c	Tissue	↓,↑^U^	[114, 137, 138]/[122]U
miR-30a	Tissue	↓,↓^B^	[24, 139]/[123]B	miR-30b b	Tissue,Urine	↓^b^,↑^b^,↓^U^	[30-32]/[140]U
miR-30e-5p	Tissue	↓	[110, 141]	miR-31	Tissue	↓	[30, 142, 143]
miR-34a	Tissue	↓,↓^B^	[76, 144]/[145]T&B	miR-92a	UrineBlood	↑^U^,↓^B^	[126]U/[135, 146]B
miR-93	Tissue,Urine	↑,↑^U^	[18, 27, 42]/[39]U	miR-96	Tissue,Urine	↑,↑^U^	[9, 125]/[116]T&U/[147]U
miR-99a	Tissue,Urine, Blood	↓,↓^U^,↓^B^	[9, 28]/[116]T&U [140]U/[117]B	miR-100	TissueUrine,Blood	↓,↓^U^,↓^B^	[9, 27, 28, 33]/[40]U/ [117, 146]B
miR-101	Tissue	↓	[114, 148, 149]	miR-106a	Tissue,Blood	↑,↑^B^	[27]/[150]B
miR-106b	Tissue	↑	[27, 33, 151]	miR-122	Tissue,Blood	↓,↓^B^	[127]B/[152]
miR-124	Tissue	↓	[153, 154]	miR-125a	Tissue,Urine	↓,↓^U^	[9, 24, 28, 33, 42, 125]/[155]U
miR-125b	Tissue,Urine	↓,↓^U^	[9,24,27,28, 33, 42, 125]/ [116]T&U/[126, 155]U	miR-126	Tissue,Urine	↓,↑^U^	[42, 131]/[155, 156]U
miR-127-3p	Tissue	↓	[27, 112, 141]	miR-130a	Tissue,Blood	↓,↑^B^	[127]B/[33]
miR-130b	Tissue	↑	[27, 29, 33, 51]	miR-133a	Tissue,Urine	↓,↓^U^	[9, 27, 28, 37, 111]/[116]T&U
miR-133b	Tissue,Urine	↓,↓^U^	[9, 28, 37, 111, 115]/ [116]T&U	miR-135b	Tissue,Urine	↑,↑^U^	[27]/[40]U
miR-138 c	Tissue	↑^c^,↓^c^	[27, 37, 38]	miR-139-5p	Tissue, Blood	↓,↑^B^	[27, 33, 111, 157]/[10]B
miR-140-3p	Tissue	↓	[111, 141]	miR-141	Tissue,Urine	↑,↓^U^	[9, 27, 28, 33]/[41]U
miR-142-3p	Urine,,Blood	↓^U^,↑^B^	[126]U/[150]B	miR-143	Tissue,Urine,Blood	↓,↓^U^,↓^B^	[27,28,37, 42,114, 125,131,158, 159]/[116]T&U/[126]U/[146]B
miR-144-5p	Tissue,Blood	↓,↑^B^	[10]B/[160]	miR-145	Tissue,Urine	↓,↓^U^	[9, 24, 27, 28, 37, 42, 114, 125, 158, 159] /[161, 162]U
miR-146a-5p	Urine,Blood	↑^U^,↓^B^	[127] B/[163]U	miR-148a	Tissue,Urine	↓,↓^U^	[126]U/[164]
miR-149	Tissue,Urine	↑,↑^U^	[27]/[126]U	miR-150	Tissue	↓	[24, 42]
miR-152	Tissue,Urine, Blood	↓,↓^U^,↑^B^	[24, 112]/[74]U/[123]B	miR-155 d	Tissue,Urine	↑,↑^Ud^,↓^Ud^	[131, 165]/[41, 166]U
miR-182	Tissue,Urine	↑,↑^U^	[9, 27, 37, 114]/[156]U	miR-182-5p	Tissue	↑	[115, 167]
miR-183	Tissue,Urine	↑,↑^U^	[9, 114, 125]/[116]T&U/ [147]U	miR-186	Tissue	↓	[111, 168]
miR-187	Tissue,Urine	↑,↑^U^	[27]/[126]U	miR-192	Urine,Blood	↓^U^,↑^B^	[10]B/[41]U
miR-194	Tissue,Blood	↓,↓^B^	[117]B/[169]	miR-195	Tissue	↓	[9, 24, 170]
miR-196a	Tissue	↑	[37, 114]	miR-199a-3p	Tissue	↓	[27, 28, 33, 112]
miR-199a-5p	Tissue	↓	[27, 28, 112]	miR-199b	Tissue	↓	[37, 42]
miR-200a	Tissue,Urine	↑,↓^U^	[9, 27, 28, 33, 42, 120]/ [41, 161]U	miR-200b	Tissue,Urine,Blood	↑,↓^U^,↑^B^	[9, 27, 28, 37]/[135]B/[41]U
miR-200b-3p	Tissue,Urine	↑,↑^U^	[9, 27, 113, 141]/[132]U	miR-200b-5p	Tissue	↑	[27, 28, 141]
miR-200c	Tissue,Urine	↑,↓^U^	[9, 27, 28, 31, 39, 42]/ [41]U	miR-200c-3p	Tissue,Urine	↑,↑^U^	[27]/[132]U
miR-203	Tissue,Urine	↑,↓^U^	[27, 114]/[40]U	miR-204	Tissue,Urine	↓,↓^U^	[27, 37, 111]/[126, 140]U
miR-205	Tissue,Blood	↑,↑^B^	[27, 33, 120, 130]/[171]B	miR-205-5p	Tissue,Urine	↑,↑^U^	[27]/[132]U
miR-210	Tissue,Urine, Blood	↑,↑^U^,↑^B^	[27, 42]/[122]U/[172]B	miR-214	Tissue,Urine	↓,↓^U^	[27, 33, 111]/[173]U
miR-218	Tissue	↓	[111, 120, 174, 175]	miR-221	Tissue	↓	[28, 42]
miR-222 e	Tissue,Urine	↓^e^,↑^e^,^U^	[10]U/[33-36]	miR-223	Tissue	↓	[24, 112]
miR-224	Tissue	↑	[27, 114, 159]	miR-301a	Tissue	↑	[27, 29]
miR-301b	Tissue	↑	[27, 29]	miR-320a	Tissue,Blood	↓,↓^B^	[127]B/[176]
miR-328	Tissue,Urine	↓,↓^U^	[27]/[40]U	miR-338-3p	Tissue,Urine	↓,↓^U^	[27]/[126]U
miR-342-3p	Urine,Blood	↓^U^,↑^B^	[10]U&B	miR-362-5p	Tissue,Blood	↑,↑^B^	[27]/[127]B
miR-370	Tissue	↓	[111, 177]	miR-374a	Blood	↑^B^	[10, 150]B
miR-423-5p	Tissue,Blood	↑,↓^B^	[27]/[127]B	miR-424	Tissue,Urine	↓,↓^U^	[126]U/[178]
miR-429	Tissue,Urine	↑,↓^U^	[9, 27]/[41]U	miR-451	Tissue	↓	[37, 179]
miR-483-5p	Tissue,Urine	↑,↑^U^	[27]/[10]U	miR-490-3p	Tissue	↓	[9, 27, 112]
miR-490-5p	Tissue	↓	[9, 27, 115, 180, 181]	miR-497	Tissue,Blood	↓,↓^B^	[27, 182]/[117]B
miR-505	Tissue,Blood	↑,↑^B^	[27]/[117]B	miR-574-3p	Tissue,Blood	↓,↑^B^	[111, 183]/[10, 127]B
miR-625-3p	Tissue,Blood	↑,↑^B^	[27]/[10]B	miR-629-3p	Tissue,Blood	↑,↑^B^	[27]/[10]B
miR-671-3p	Tissue,Blood	↑,↑^B^	[27]/[10]B	miR-708	Tissue	↑	[7, 27, 28]
miR-1207-5p	Tissue,Urine	↓,↓^U^	[116]T&U	miR-1224-3p	Tissue,Urine	↓,↓^U^	[40]U/[74]T&U

Researches are based on ^T^ Tissue, ^U^ Urine, ^B^ Blood, ^T&U^ both Tissue and Urine, ^T&B^ both Tissue and Blood, ^U&B^ both Urine and Blood.

**Figure 2:**
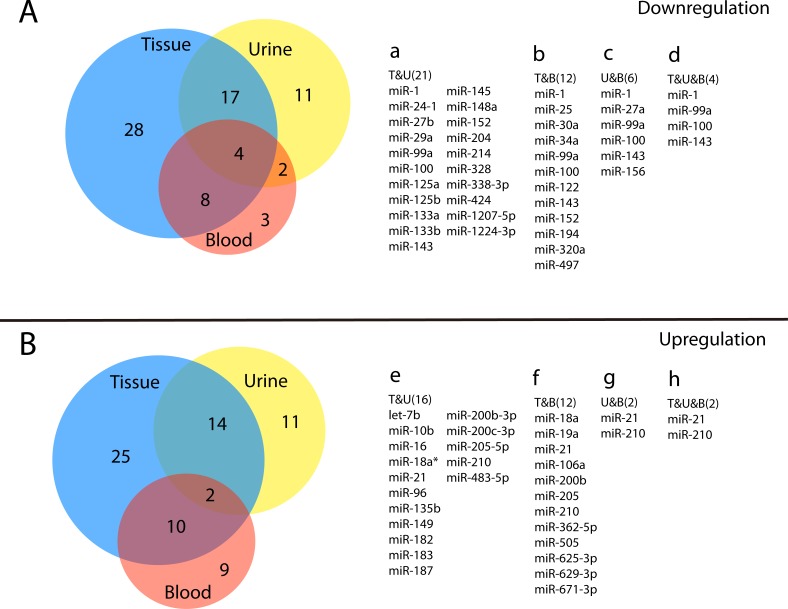
Venn diagram of multiple reported miRNAs in three biological samples 73 miRNAs are downregulated(A) and 71 are overexpressed(B). Differences of the expression exists among clinical bladder tumor tissues (blue circle), urine samples (yellow circle) and blood (red circle). 21, 12 and 6 miRNAs decrease respectively in subgroups of “Tissue and Urine” (a), “Tissue and Blood” (b) and “Urine and Blood” (c), and 16 (e), 12 (f) and 2 (g) miRNAs are correspondingly over-expressed. The overlapping part of three circles represents miR-1, miR-99a, miR-100 and miR-143 in A, and miR-21 along with miR-210 in B. T&U, Tissue and Urine; T&B, Tissue and Blood; U&B, Urine and Blood; T&U&B, Tissue and Urine and Blood.

↑, Upregulated; ↓, Downregulated; ↑or↓ without superscript are variations in bladder cancer tissue.

a: MiR15a is upregulated in urine samples according to ref.^39^ while ref.^40^ shows its downregulation;

b: MiR-30b is shown to increase in ref.^31^-^32^ and decrease in ref.^30^,^140^ (urine sample);

c: MiR-138 is upregulated according to ref.^38^. That result is in contradiction to the results of ref.^27^ and ref.^37^;

d: The level of miR-155 increases in urine supernatant meanwhile decreases in urine sediment(ref.^41^).

e: Over-expression of miRNA-222 is reported in ref.^34^-^35^ which contradicts the results of downregulation in the rest references;

Until the present, miRNA changes in bladder tumour tissues have been the most widely reported among 3 types of biological samples. Out of 118 miRNAs listed in Table 1, 111 (94.06%) have been shown to be dysregulated in clinical specimens, among which 57 (51.35%) are decreased or silenced, including miR-100, the miR-125 family, the miR-133 family, miR-143 and miR-145 [9, 27, 28]; and 51 (45.95%) miRNAs are over-expressed, such as the miR200 family (miR-200a/b/c) and the miR-130 family (miR-130b, miR-301a and miR-301b) [9, 27-29] (Table 1). The variation trends in five miRNAs have been the subjects to contradictory reports [27, 30-41]. It is not hard to see that the variations in the miRNA expression levels are not exactly the same in terms of biological sample types. The tissue and urine samples share the most members (37 miRNAs) that have the same variation trends. Twenty-one miRNAs are congruously downregulated in both tissues and urine samples (Figure 2A) while sixteen are over-expressed (Figure 2B). Compared to the studies based on tissues and urine samples, few studies have focused on the variance between urine miRNAs and circulating miRNAs. It is noteworthy that miR-1, miR-99a, miR-100 and miR-143 (Figure 2A) are similarly silenced in all three types of biological samples compared to the normal urothelia and body fluids of healthy people. However, miRNA-21 together with miR-210 are uniformly over-expressed (Figure 2B) in three types of samples. Out of all the miRNAs that are listed in Table 1, miR-30b, miR-222, miR-138, miR15a and miR155 were not chosen to be presented in Figure 2 due to the inconsistency among different studies.

Interestingly, apart from the miRNAs that share the same variation trends in different types of samples, a large number of BCa-related miRNAs show disparate changes in different sample types. For example, miR-342-3p is found to be upregulated in blood but downregulated in the patients’ urine [10]. Similarly, miR-200c is widely reported to be over-expressed in tumour tissues [9, 28, 42], while it is expressed to a lesser degree in the urine samples [41]. Since most research on bladder cancer is based on tissue samples, these differences suggest that urine or circulating miRNAs may be disparately expressed, and they may work through totally different mechanisms compared to tissue miRNAs. More research is needed to find the different expression profiles and working mechanisms of urine miRNAs as well as circulating miRNAs in patients with bladder cancer and to identify a certain miRNA or a series of miRNAs, if possible, as non-invasive biomarkers for the screening and diagnosis of this disease.

## ABERRATION IN THE MIRNA BIOGENESIS PROCEDURE IN BLADDER CANCER

Since the expression process of each miRNA should go through the entire miRNA biogenesis pathway, it is easy to deduce that the impaired miRNA biogenesis and regulation procedure plays a vital role in the above-mentioned aberration and malfunction of miRNAs in bladder cancer [14]. Previous studies have shown that in bladder cancer, various factors can interfere with multiple levels of miRNA biogenesis. By reviewing the updated literature in this area, we are the first to give a comprehensive outline about how the aberrance of miRNA biogenesis results in the dysregulation of miRNAs in bladder cancer, which is classified by the different steps and molecules in the miRNAs biogenesis pathway. We also gather evidence to show that the disorders of both miRNA expressions and the biogenesis pathway are associated with bladder carcinogenesis as well as tumour progression.

### Malfunctions of miRNA coding genes related to bladder cancer

The biogenesis pathway of BCa-related miRNAs initiates with the transcription of miRNA coding genes. The aberrations at the genetic/chromosomal level would therefore become the very first factor that can alter miRNA expressions. The miRNA coding genes could be impaired by either genetic variations or epigenetic modifications, or they could be influenced by transcription factors and host genes.

#### Genetic variations

The single-nucleotide polymorphism (SNP) is a rather common type of genetic variation in cancer. The SNPs in miRNA coding genes have been validated to directly alter the transcription and maturation of miRNAs [43], and they may be associated with the cancer risk and prognosis [44]. A study that was performed by Wang *et al*. reveals that the SNP rs2910164 in the miR-146a gene is strongly related to the biological characteristics of bladder cancer, and the rs2910164 C allele, markedly enhances the expression level of miR-146a (a tumour suppressor) in bladder cancer cells compared to the G version and therefore inhibits cell proliferation. An additional study shows that patients carrying allele C are endowed with a significantly decreased bladder cancer risk (a 20% decrease compared to the G allele) as well as a reduced risk of recurrence [45]. Analogously, a SNP rs11671784 G/A variation in the miR-27a gene decreases the miR-27a level and reduces the chemo-sensitivity of bladder cancer [46].

Remarkably, other types of genetic variations, such as gene deletions or point mutations, have also been shown to impact miRNA transcription and then lead to the dysregulation of miRNA expression, which may be associated with the clinical processes of various cancers [47, 48]. In bladder cancer, Veerla *et al.* report that miR-31, which is located in 9p21, is found to be homozygously deleted in patients, and this gene deletion may be related to the downregulation of its expression [49]. To summarize, the gene variations of a certain miRNA may not only alter the expression level of itself but will also be of great significance to the biological features of bladder cancer, such as tumour progression, metastasis, recurrence and chemo-resistance. We have noted that in comparison to the large number of studies based on SNPs, research on other types of genetic variations in BCa such as gene deletions or point mutations have not been widely performed. Thus, more in-depth studies are needed to determine how different types of genetic variations alter the expression of BCa-related miRNAs and what their biological significance is.

#### Transcription factors

In addition to the gene variations, numerous transcription factors inside the nucleus would influence miRNA gene transcription. One of the most convincing pieces of evidence is that NF-κB can enhance the expression of miR-130b. In bladder cancer, the nuclear factor-kappa B (NF-κB) can be promoted by MLK3 [50] and is shown to induce the expression of miR-130b, an oncogenic miRNA, by directly binding to the promoter region of the miR-130b gene [51]. In addition, Snail-1, which is regulated by the Akt/GSK-3β pathway in BCa, can transcriptionally promote the expression of miR-21 and miR-29 [52, 53]. Some other popular transcription factors are also reported to alter the expression of different miRNAs through the regulation of their transcription (Table 2). Interestingly, most of those miRNAs play an oncogenic role in bladder cancer, which indicates that the regulation by transcription factors could be an important part of the oncogenic miRNA biogenesis pathway and may be relevant to bladder tumourigenesis.

**Table 2:** Common transcription factors regulating miRNA gene transcription in bladder cancer

Transcription factors	Regulated miRNAs	TFs binding sites	MiRNA functions	References
HIF-1α	miR-145	MiR-145 promoter	Promoting apoptosis	[184]
Snail-1	miR-21, miR-29	Unclear	Promoting metastasis	[53]
p53	miR-200 family	5’ promoters of the miRNAs	Inhibiting EMT *via* decreasing ZEB1/2	[185]
NF-κB	miR-130b	MiR-130b promoter	Promoting cell proliferation, invasion and migration	[51]
TWIST1	miR-200 family, miR205	MiR-200 and miR-205 promoters	Inhibiting EMT *via* decreasing ZEB1/2	[186]
PTEN	miR-21,miR-19a, miR-25	MiRNAs promoters	Oncogenic microRNAs	[187]
p63 ΔNp63α	miR-205	Highly conserved regulatory region upstream of the miR-205 start site	Inhibiting EMT *via* decreasing ZEB1/2	[65]
VHL	miR-210	Unclear	Promoting cell growth and migration	[187, 188]

TFs, transcription factors; HIF-1α, Hypoxia-inducible factor 1α; Snail-1, zinc finger protein SNAI1; ΔNp63α, p63 isoform protein; VHL, von Hippel-Lindau tumor suppressor; EMT, Epithelial-mesenchymal transition.

#### Epigenetic modifications

Epigenetic modifications of miRNA genes have become a research hotspot in recent years. Although individual miRNAs may be either downregulated or over-expressed, depending on their function, previous research still shows that the overall miRNA expression level is reduced in tumours [54]. Despite our vague understanding of this phenomenon, scientists still believe that epigenetic modifications, including DNA hypermethylation and histone modification, play a key role [55-57]. In bladder cancer, miRNA silencing caused by epigenetic modifications has been widely recognized. Takashi *et al*. use 5-aza-dC to demethylate two bladder cancer cell lines and find that up to 146 silenced miRNAs are upregulated after the treatment. An additional study shows that in comparison to normal bladder tissues, the methylation level of miRNA genes in bladder cancer tissues is significantly upregulated (>15%) [20]. The expression of miR-137, an important bladder cancer suppressor [58], is negatively correlated with its methylation in bladder tumour tissue specimens, which indicates that bladder tumourigenesis may be associated with the silence of miRNAs caused by DNA hypermethylation [20]. To gain a better understanding of the epigenetic regulations in BCa, we list the major miRNAs for which the expressions are reportedly regulated *via* epigenetic modifications in Table 3.

**Table 3:** Epigenetic modifications of miRNAs in bladder cancer

MiRNAs	Epigenetic modifications	Location	Targets	MiRNA functions	↑/↓	Clinical significances	Sample types	References
miR-9-3	CGI hypermethylation, HM	15q26.1	-	Tumor suppressor	↓	Potential biomarker	Tissue, Urine, Cell lines	[20, 74]
miR-137	CGI hypermethylation, HM	1p21.3	PAQR3	Tumor suppressor	↓	Potential biomarker	Tissue, Urine, Cell lines	[20, 58]
miR-124-2	CGI hypermethylation, HM	8q12.3	UHRF1	Tumor suppressor	↓	Potential biomarker	Tissue, Urine, Cell lines	[20, 153]
miR124-3	CGI hypermethylation, HM	20q13.33	UHRF1	Tumor suppressor	↓	Potential biomarker	Tissue, Urine, Cell lines	[20, 153]
miR-1224-3p	Hypermethylation of CpG shore> CGI	3q27.1	-	Inhibiting cell growth	↓	Related to metastasis and poor prognosis	Tissue	[74]
miR-152	CGI hypermethylation	17q21.32		Inhibiting cell growth	↓	Potential biomarker	Tissue	[74, 189]
miR-200a/b	Promoter CGI hypermethylation, H3K9Ac, H3K27me3	1p36.33	ZEB1/ZEB2	Regulating EMT	↓	Predicting prognosis	Cell lines	[186, 189]
miR-200c	Promoter CGI hypermethylation, H3K9Ac, H3K27me3	12p13.31	ZEB1/ZEB2	Regulating EMT	↓	Associated with progression of T1 stage tumor	Cell lines	[186]
miR-205	Promoter CGI hypermethylation	1q32.2	ZEB1/ZEB2	Regulating EMT	↓	Predicting prognosis	Tissue	[186]
miR-10a	Gene hypermethylation	17q21.32	-	Unclear	↓	Potential biomarker	J82 cell lines	[189]
miR-193a-3p	Gene hypermethylation	17q11.2	SRSF2/PLAU/HIC2	Related to chemo-resistance	↓*	Chemo-resistance due to the hypermethylation	5637 and H-bc cell lines	[190]
miR-34a	Promoter CGI hypermethylation	1p36.22	HNF4G	Tumor suppressor	↓ in MIBC	Chemo-sensitivity enhanced by demethylation	Tissue, Cell lines	[72, 76]
mir-516a	CGI hypermethylation	19q13.42	-	Unclear	Uncelar	Potential biomarker	RT112 and RT4 cell lines	[74]
mir-517a	Gene hypermethylation	19q13.42	AREG/BCLAF1	Accelerating apoptosis	↓	Demethylation as a potential treatment	Boy and T24 cell lines	[191]
miR-551a	CGI hypermethylation	1p36.32	-	Tumor suppressor	Uncelar	Potential biomarker	RT112 and RT4 cell lines	[74]
miR-126	Host gene EGFL7 is regulated by HM	9q34.3	ADAM9	Tumor suppressor	↓	New anti-cancer mechanism of epigenetic medicines	Tissue, T24 cell lines	[66, 192]
miR-127	CGI hypermethylation, Histone deacetylation	14q32.31	BCL6	Tumor suppressor	↓	Demethylation and deacetylation as potential therapies	Tissue, T24 cell lines	[56]
miR-99a	Long range epigenetic regulation	21q21.1	FGFR3	Inhibiting cell proliferation, migration and invasion	↓ in low-grade NMIBC	Specifically characterizing low-grade tumor	Tissue, T24 and EJ Cell lines	[18, 193]
miR-100	Long range epigenetic regulation	11q24.1	FGFR3	Decreasing cell viability in 3-D growth	↓ in low-grade NMIBC	Specifically characterizing low-grade tumor	Tissue, RT4 cell lines	[18, 194]

HM, histone modifications; CGI, CpG Island; H3K9Ac, H3 lysine 9 acetylation; H3K27me3, H3 lysine 27 trimethylation; EMT, epithelial-mesenchymal transition; MIBC, muscle invasive bladder cancer; NMIBC, non- muscle invasive bladder cancer.

*Downregulated in chemo-sensitive bladder cancer cell lines but overexpressed in chemo-resistant cell lines.

**Figure 3:**
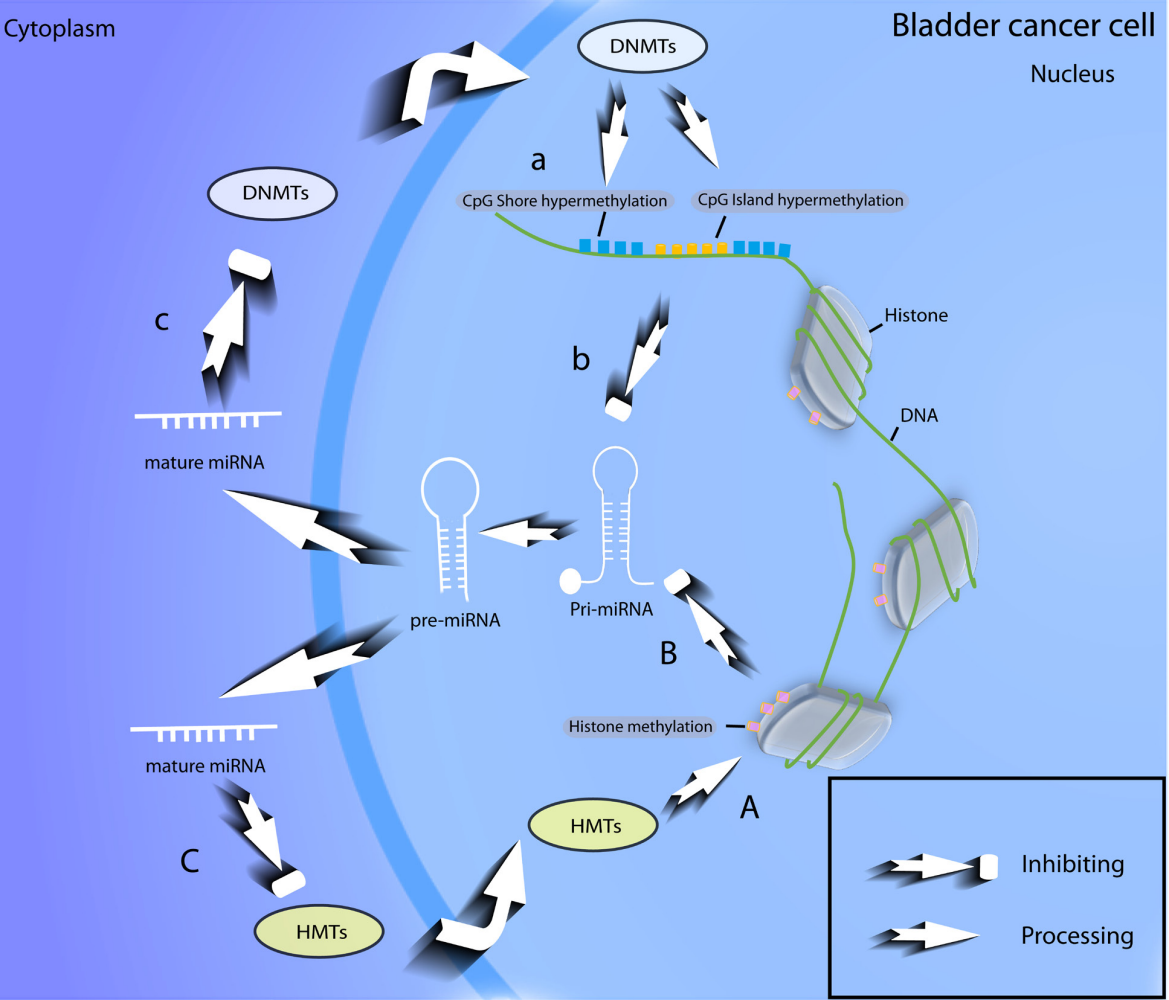
Feedback loop of epigenetic modifications of miRNAs in BCa With the help of DNMTs and HMTs, miRNA coding genes (a) and related histones (A) can be modified epigenetically, thus anti-cancer miRNAs in urothelial cells are silenced (b, B); Some miRNAs can in turn suppress the expression level of DNMTs (c) and HMTs (C). DNMTs, DNA methyltransferases; HMTs, histone methyltransfereases.

From above knowledge about the epigenetic regulations of miRNA genes, we can see that it is the epigenetic modifications of the anti-cancer miRNAs, especially DNA hypermethylation, that inhibit the formation of primary transcripts and further induce bladder tumourigenesis. However, miRNAs may conversely adjust the core components of the epigenetic modification process. Various BCa-related miRNAs, such as miR-148a, miR-143 and miR-101, are found to have a function in inhibiting DNA methyltransferase (DNMTs) [59, 60] and EZH2 (Histone H3K9 methyltransferase) [61, 62] and are thus, to some extent, involved in epigenetic modifications. The interactions between miRNA and epigenetic modifications can be summarized as a feedback loop (Figure 3), which is a fundamental mechanism of the disorder in the miRNA biogenesis of bladder cancer.

#### Host genes

MiRNAs can be divided into two groups, intergenic miRNAs and intragenic miRNAs, according to their genomic localizations. Intergenic miRNAs are considered as independent transcriptional units while intragenic miRNAs are embedded in other genes; those genes are known as the host genes for the miRNAs [63]. The transcription of intragenic miRNAs is in parallel with their host genes [64]. In bladder cancer, the transcription of some miRNAs is dysregulated due to the changes in their host genes. MiR-205 is located within the LOC642587 gene, which is known as miR-205HG (miR-205 host gene). MiR-205 and miR-205HG share the same gene promoter. ΔNp63α (an isoform of p63) can bind to a highly conserved region upstream of the miR-205 start site and increase the transcription of both miR-205HG and miR-205 in bladder cancer cell lines. The knockdown of ΔNp63α in BCa cell lines directly attenuates the binding of RNA Pol II to the miR-205HG promoter and reduces the transcription of both miR-205 and miR-205HG [65]. In addition, EGFL7 is the host gene of miR-126 in bladder cancer tissue and cell lines. The downregulation of miR-126 in BCa is caused by histone modifications of EGFL7 [66]. The above research shows that the upregulation or downregulation of miRNAs transcription in bladder cancer could be modulated indirectly, namely *via* the host genes.

### Disorder of miRNA transcript processing in bladder cancer

As mentioned above, the pri-miRNA is cleaved by the Microprocessor complex into pre-miRNA. A malfunction at this step would also participate in the aberration of miRNAs and bladder carcinogenesis. One of the most important studies is about the star molecule, p53. TP53 is a vital tumour-suppressor gene whose mutations are deeply embedded in the tumourigenesis of almost all types of malignancies, including bladder cancer. To put it briefly, p53 protein is coded by the TP53 gene, which works to protect cells from the carcinogenesis induced by the accumulation of oncogene mutations [67]. In urinary bladder carcinoma, the disorder of the TP53 gene and its downstream pathways are usually related to muscle invasion, higher stage metastasis, and recurrence as well as poor prognosis [68, 69]. In addition, p53 has been shown to be a key factor in regulating the miRNA biogenesis pathway. The processing of pri-miR-34a has been shown to be promoted by p53 with the help of SIRT1. p53 can interact with DROSHA, thereby promoting the anti-cancer pri-miRNAs to convert into pre-miRNAs [70, 71]. This p53/miR-34a axis also has significant functions in bladder carcinogenesis, such as inhibiting invasion, decreasing recurrence, preventing metastasis and anti-angiogenesis [72-74]. After polyphenol treatment, the proliferation of bladder cancer cells is inhibited by enhancing the function of the p53/miR-34a axis [75]. This result is consistent with what occurs in bladder cells after cisplatin treatment [76], which suggests that different drugs may repair the impaired miRNA biogenesis pathway in bladder cancer cells to some extent by enhancing the expression of p53-mediated miRNAs.

The disorder of pri-miRNA processing caused by the aberration of the Microprocessor complex (DROSHA/DGCR8) will be discussed separately in the text that follows.

### Defective maturation of BCa-related miRNAs

After the synthesis of precursor miRNAs, the RNAs are further processed to generate mature molecules. As a matter of course, the defects at this step result in changes in the BCa-related miRNA expression. The let-7 family, which is known as a group of tumour suppressor miRNAs, could prevent tumourigenesis and cancer development by suppressing the expression of oncogenes and controlling the cell cycle [77, 78]. In bladder cancer, gefitinib exerts its anti-cancer effect by upregulating let-7 [79]. The expression of let-7 is regulated by Lin28 protein, and the interaction of the two proteins forms a regulatory circuit [80, 81]. Other researchers have noted that the over-expression of Lin28 A or B in human tumours is correlated with the downregulation of let-7 and may be associated with poor prognosis and decreased survival rate in cancer patients [82, 83]. The studies mentioned above indicate that the Lin 28 protein is involved in carcinogenesis and the clinical processes of cancer due to its inhibiting effect on the expression of let-7. In urinary bladder cancer, Lin28 can prohibit the conversion from pre-let-7 to mature let-7. Li *et al*. found that with the over-expression of Lin 28, the pre-let-7a in bladder tumour tissues is correspondingly upregulated, with a significant downregulation of mature let-7a [84]. Moreover, the expression level of pre-let-7a is inversely associated with the pathological grades of urothelial carcinoma, which demonstrates a probable way in which the defective maturation of pre-miRNAs influences the pathological features of bladder cancer. Park *et al*. discover that in bladder cancer cells, the loss of the histone variant, macroH2A1, can significantly suppress the expression of mature let-7 *via* the upregulation of Lin 28 [85]. This result reconfirms what Lin *et al*. found in their study. According to a study by Thornton *et al*., Lin 28 inhibits the formation of mature let-7a by binding to the terminal loop region of pre-let-7a so that the Dicer enzyme could not further process it, and finally, the precursor miRNA would be degraded [80]. Once Lin28 was bound to pre-let-7a, it could recruit uridylyltransferases to add an oligouridine tail to pre-let-7, which could then prevent pre-let-7 from being subjected to DICER processing [86].

In other words, the defective maturation of pre-let-7a would directly induce the low expression of let-7a and therefore cause the abnormal differentiation of bladder urothelial cells. Apart from let-7a, however, limited miRNAs are reported in bladder cancer to be dysregulated at the “precursor to mature molecule” level. Hence, future studies about the molecular mechanisms of bladder tumours may focus on the aberrant maturation of different pre-miRNAs and on their interaction with various proteins.

### Core components of miRNA biogenesis machinery in the context of bladder cancer

Multiple proteins participate in the miRNA biogenesis network, among which the Microprocessor (DROSHA/DGCR8), XPO5 and DICER are widely studied. The different roles of these components are shown in Figure 1. If the biological processing of miRNAs is compared to a production line, those proteins would become miRNA biogenesis machinery, whose dysregulation and malfunction will profoundly alter the output of the products-miRNAs and further play a role in bladder tumourigenesis and cancer progression.

#### Microprocessor complex

DROSHA and DGCR8 make up the Microprocessor complex of miRNA biogenesis and can process primary miRNAs into precursor miRNAs [87, 88]. Studies reveal that the aberrant expression of DROSHA is closely related to carcinogenesis because of the significant variations that it causes in miRNA expression [89]. This aberrance will promote the proliferation, metastasis and invasion of tumour cells, thus eventually accelerating the clinical progression of cancer [90]. In bladder tumour tissue specimens from clinical patients, a remarkable increase in expression is observed in both the DROSHA protein and its mRNA. The over-expression of DROSHA is then shown to be associated with poor bladder cancer prognosis [19, 91]. Catto notes that in comparison with healthy controls, the obvious disorder of miRNA expression in patients with bladder cancer is related to the transcriptionally over-expressed DROSHA in bladder tumour tissues [18]. In other words, the upregulation of DROSHA will directly lead to changes in the miRNA expression profile, and, as a result, affect the clinical development of the disease. Besides, the genetic variations in the DROSHA gene may also be a reason for its overexpression. An SNP rs10719T>C polymorphism located in the DROSHA gene 3’ UTR could regulate the binding activity of miR-27b. The alteration from the T allele to the C allele helps the gene to get rid of miR-27b control, leading to the enhanced expression of the DROSHA enzyme and a significantly increased risk of bladder cancer [92]. This research also illustrates that the high expression of DROSHA in bladder cancer and the variation in miRNA expression may involve feedback loop regulation, and they may cooperatively participate in bladder tumourigenesis.

Unlike Lin 28 protein, whose upregulation specifically alters a certain miRNA (let-7), the upregulation of DROSHA is not associated with specific miRNAs. Instead, miRNAs are globally dysregulated due to the over-expression of DROSHA protein in bladder cancer [18]. Perhaps the primary reason is that DROSHA takes part in the common step (“pri-miRNA cleavage”) of the biogenesis pathway of each miRNA. Although it is easy to understand that the upregulation of the DROSHA protein can increase the expression of oncogenic miRNAs in bladder cancer, the question as to whether upregulated DROSHA also plays a role in terms of numerous downregulated/silenced miRNAs remains to be answered.

Unlike DROSHA, studies on the changes in DGCR8 in bladder cancer are still very limited. We only know that DGCR8 is also over-expressed in bladder cancer tumour tissues, but it is downregulated in the normal urothelia of patients with bladder cancer [18]. The SNPs in the DGCR8 gene (rs2073778 and rs720012) are associated with the risk of progression and the muscle invasion of NMIBC [93], suggesting that the malfunction of DGCR8 also shows its special clinical significance in bladder cancer.

#### XPO5

XPO5 (also known as Exportin 5) works as a transporter to pump pre-miRNA into the cytoplasm [87]. A large scale of dysregulated miRNAs in the normal urothelia of bladder cancer patients is strongly related to the high expression of XPO5 [18], and the XPO5 levels in high grade bladder cancer and invasive bladder cancer are significantly higher than in the levels of low grade and non-invasive bladder cancer. Silencing XPO5 inhibits proliferation and promotes the apoptosis of bladder cancer cells [91]. Similar to the Microprocessor complex, the upregulation of XPO5 also occurs at the transcriptional level [91]. We speculate that the over-expression of XPO5 might accelerate the transportation of tumour-promoting miRNAs to the cytoplasm and therefore alter the expression profile of miRNAs in bladder cancer. More research is needed in terms of the changed miRNA expression caused by the dysregulation of XPO5.

#### DICER

DICER is a type of RNAse III enzyme, and it can process the pre-miRNA into a mature duplex [87]. The altered expression level of DICER is reported in various tumours, and its dysregulation is considered to be tightly associated with variations in the miRNA expression profile, which can further mediate carcinogenesis and the poor outcome of malignancies [94, 95]. Confusingly, DICER mRNA and protein have been shown to be upregulated in BCa only by some researchers, and this upregulation is shown to have close relations to poor clinical prognosis for bladder cancer [19, 91]. However, for the rest of the studies, totally conflicting results are reported. Those studies reveal low expression for DICER mRNA in bladder cancer tissues [18, 96], and this low expression is associated with the more malignant phenotypes of the disease [97]. In BCa, PKC-α is a key factor that modulates the expression of DICER. The reduction of PKC-α can induce lower DICER expression at both the mRNA and protein level and lead to the apoptosis of bladder cancer cells [98]. In contrast to PKC-α, the miR-18a in bladder cancer T24 cells can downregulate the transcription of DICER mRNA [99]. A recent study shows that the change of DICER in BCa may alter the miRNA expression and be involved in the development of the disease in that the attenuated DICER expression can induce a decreased level of invasion-associated miRNAs in BCa such as in the miR-200 family, miR-205, miR-31, miR-148a, miR-149 and miR-106b and further potentiate the invasion of bladder cancer cells [97]. There is no doubt that the DICER expression change in bladder cancer mediates a variation of the miRNA expression profile and plays a rather important part in the development of this urinary carcinoma. However, the real variation trend for DICER in BCa and the way in which it works towards the disorder of miRNAs are still unclear.

In addition to the molecules mentioned above, some other important proteins, such as TRBP (that works together with DICER) and AGO (a component of miRISC), are also involved in the miRNA biogenesis machinery (Figure 1). Further studies on the roles of those molecules in the aberrantly expressed miRNAs of bladder cancer will definitely promote a better understanding of this field.

### Dysfunction of the miRNA-mRNA binding process in bladder cancer

It has been confirmed that mature miRNAs target the miRNA-binding sites located in the 3’ UTR of the mRNAs [100]. This type of binding is commonly considered as the final step of the whole miRNA biogenesis procedure [11, 21]. One possible mechanism of the malfunction in the miRNA-mRNA binding process is that the variations in the miRNA binding sites of the target mRNAs disrupt the binding activity so that those mRNAs are set free from the miRNA controls. This mechanism is extensively applicable to miRNAs in bladder cancer. The over-expression of the oncogene HOXB5, for instance, can be inhibited by miR-7 in urothelial cells, and SNP 1010A/G is located in the miR-7 binding site of HOXB5 gene. A previous study shows that the existence of the G allele can significantly weaken the binding activity of miR-7 and therefore protect HOXB5 mRNA from the suppression of miR-7. The resulting high expression of HOXB5 leads to higher stages and higher grades of bladder cancer [101]. Similar variations in miRNA binding sites are also reported for the 3'UTR of PARP1, TP63 and ITGB5 mRNA [102-104]. In brief, a large amount of evidence shows that even after the mature miRNAs are produced, the genetic variations on the miRNA binding sites could also impair the function of miRNAs and further have a deep impact on the tumourigenesis and progression of bladder cancer.

Additionally, this focus will soon become a hotspot for miRNA researchers after the concept of competing endogenous RNAs (ceRNAs) is put forward. CeRNAs have a function in inhibiting miRNA-mediated gene silencing [105]. Thus, the dysregulations of miRNAs caused by ceRNAs are also included in the disorders of the final step in the miRNA biogenesis pathway. It is reported that the lncRNA H19 and circRNA MYLK could act as ceRNAs in bladder cancer and bind competitively with miRNA-29a-3p so that the function of mature miRNA-29a-3p is inhibited [106]. From the available literature, we infer that in bladder cancer, some lncRNAs as well as circRNAs can work as ceRNAs and participate in the regulation of miRNAs on the miRNA-mRNA binding level. The roles that various lncRNAs and circRNAs may play in the dysregulation of miRNAs in bladder cancer would no doubt be a valuable focus of further research.

## CONCLUSIONS AND PROSPECTIVE

**Figure 4:**
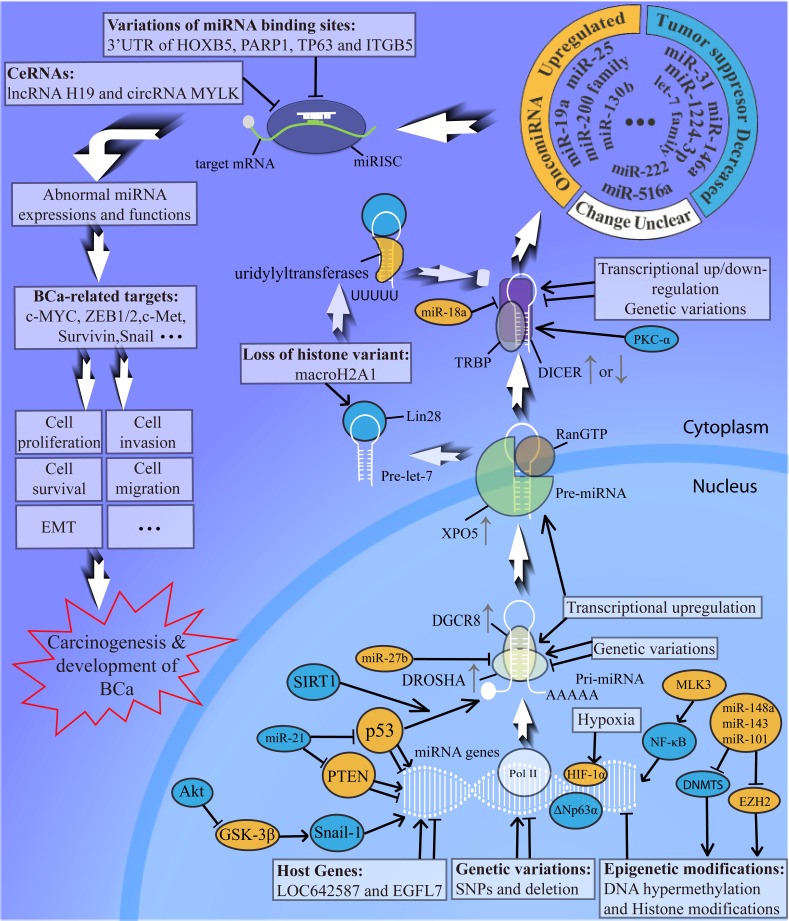
Abnormal miRNA biogenesis in bladder cancer development Abnormalities of miRNA biogenesis in BCa appear at multiple levels of the whole procedure. Genetic variations, epigenetic modifications, different transcription factors, the host genes as well as hypoxia alter the transcriptions of pri-miRNA; With the help of SIRT1, p53 accelerates the processing of pri-miRNAs (like pri-miR-34a); Microprocessor Complex and XPO5, which are transcriptionally upregulated, lead to wide aberrance of miRNAs in BCa; PKC-α, miR-18a and genetic variations induce the dysregulation of DICER and the transcriptional dysregulation of DICER will affect pre-miRNA cleavage in BCa; Loss of macroH2A1 in bladder cancer promote Lin28 to selectively bind to pre-let-7 so that pre-let-7 won't be processed by DICER; The abnormalities at different steps cause the upregulation of oncomiRNAs and the downregulation of tumor suppressors, but the changes of some miRNAs in BCa are still unclear; The variations of miRNA binding sites and ceRNAs lead to malfunction of miRNAs by inhibiting the miRNA-mRNA combination in bladder cancer; Abnormal miRNA expression and function modulate the downstream targets and signaling pathways and eventually lead to carcinogenesis and development of bladder cancer. PTEN, phosphate and tension homology deleted on chromosome ten; Akt, serine/threonine kinase; MLK3, mixed lineage kinase 3; PKC-α, protein kinase C-α; SIRT-1, Sirtuin-1; ZEB1/2, zinc finger e-box binding homeobox 1/2.

In bladder cancer research, numerous miRNAs have been recognized to be aberrantly expressed, and their downstream target genes are partially discussed [18, 24, 30]. In our review, we summarize the latest and most complete expression changes of miRNAs in bladder cancer, and we compare the differences in altered miRNAs among various biological samples. Through our literature review, we show that the cancer-related miRNA expression profiles in tumour tissues, urine and blood samples are all remarkably changed compared to BCa-free people. Variations in miRNA expressions are not identical among these three types of samples (Figure 2). Moreover, our review is the first to outline the dysregulations in the entire miRNA biogenesis process and bladder cancer development at different steps, and the abnormalities of miRNA biogenesis in BCa development are portrayed in detail in Figure 4. We note that when tracing them to their cause, the altered expressions and malfunctions of miRNAs in bladder cancer are closely related to the abnormalities in one or multiple links or factors of the whole miRNA biogenesis pathway. We also have reasons to believe that the dysregulation of a certain miRNA in bladder cancer is a result of multiple disorders in its biogenesis and functioning procedure. For example, the downregulation of the tumour suppressor miR-34a in tumour tissue samples of BCa is associated with both epigenetic regulation and the malfunction of the p53/miR-34a axis, and it may also be related to the variations in Microprocessor and DICER. Some variations such as epigenetic modifications and the abnormal Microprocessor complex can be conversely regulated by different miRNAs. In conclusion, the abnormal miRNA biogenesis in bladder cancer development might be a key mechanism in the aberrance of miRNA expressions and functions in BCa, and the dysregulated miRNAs will target the downstream signalling pathways such as ZEB1/2 and Survivin; they will eventually have a profound impact on the development of bladder cancer [84, 107-109] (Figure 4).

Due to the urgency of updating the diagnosis methods as well as treating bladder cancer, more investigators are likely to conduct research in the field of BCa-related miRNAs. Accordingly, the altered urine and blood miRNAs may be utilized as specific and non-invasive diagnostic biomarkers. To further illustrate the mechanisms of the abnormal miRNA biogenesis, more detailed and systematic experiments are required. Last but not least, the disorders in the whole miRNA biogenesis procedure may become potential targets for treating urinary bladder carcinoma. Hence, targeting drugs as well as the epigenetic chemicals that could repair the aberrations to some extent may pave the way to new bladder cancer therapies.
